# The role of connexin proteins and their channels in radiation-induced atherosclerosis

**DOI:** 10.1007/s00018-020-03716-3

**Published:** 2021-01-03

**Authors:** Raghda Ramadan, Sarah Baatout, An Aerts, Luc Leybaert

**Affiliations:** 1 Radiobiology Unit, Belgian Nuclear Research Centre (SCK CEN), Mol, Belgium; 2 Department of Basic and Applied Medical Sciences, Physiology group, Ghent University, Ghent, Belgium; 3 Department of Molecular Biotechnology, Ghent University, Ghent, Belgium

**Keywords:** Ionizing radiation, Atherosclerosis, Intercellular communication, Bystander effect, Connexin, Gap junction, Hemichannels

## Abstract

Radiotherapy is an effective treatment for breast cancer and other thoracic tumors. However, while high-energy radiotherapy treatment successfully kills cancer cells, radiation exposure of the heart and large arteries cannot always be avoided, resulting in secondary cardiovascular disease in cancer survivors. Radiation-induced changes in the cardiac vasculature may thereby lead to coronary artery atherosclerosis, which is a major cardiovascular complication nowadays in thoracic radiotherapy-treated patients. The underlying biological and molecular mechanisms of radiation-induced atherosclerosis are complex and still not fully understood, resulting in potentially improper radiation protection. Ionizing radiation (IR) exposure may damage the vascular endothelium by inducing DNA damage, oxidative stress, premature cellular senescence, cell death and inflammation, which act to promote the atherosclerotic process. Intercellular communication mediated by connexin (Cx)-based gap junctions and hemichannels may modulate IR-induced responses and thereby the atherosclerotic process. However, the role of endothelial Cxs and their channels in atherosclerotic development after IR exposure is still poorly defined. A better understanding of the underlying biological pathways involved in secondary cardiovascular toxicity after radiotherapy would facilitate the development of effective strategies that prevent or mitigate these adverse effects. Here, we review the possible roles of intercellular Cx driven signaling and communication in radiation-induced atherosclerosis.

## Introduction

Cardiovascular disease (CVD) is the leading cause of morbidity and mortality worldwide, with 31% of all global deaths in 2016, according to the World Health Organization (WHO). The most common causes of CVD morbidity and mortality are myocardial infarction, stroke, coronary artery disease, and congestive heart failure [1]. Atherosclerosis is considered the major underlying cause of CVD development [2]. The progression of atherosclerosis and the risk of CVD are influenced by the presence of a combination of risk factors, such as dietary factors, tobacco use, physical inactivity, hypertension, age, gender, hyperlipidemia, and genetic predisposition [3]. Growing evidence indicates that exposure to ionizing radiation (IR) is also associated with an increased risk of CVD [4–12].

The medical use of IR plays a key role in cancer treatment with about 50% of cancer patients receiving radiotherapy for curative and/or supportive therapy during the course of their treatment [13]. Incidental IR exposure to the heart and large arteries occurs during radiotherapy for thoracic malignancies such as breast cancer, head and neck cancer, Hodgkin's lymphoma, and esophageal cancer [14]. Large-scale epidemiological studies have established a link between high and medium doses of IR exposure (> 0.5 Gy) and the risk for CVD [5, 9, 11, 15–17]. In addition, meta-analyses of epidemiological studies, and other experimental studies suggest that even low radiation doses (< 0.5 Gy) can generate cardiovascular morbidity [7, 9, 18–23].

Radiation treatment is known to cause cellular effects such as oxidative stress, DNA damage, cellular Ca^2+^ overload, apoptosis, premature cell senescence and promotes inflammation which may induce vascular endothelium damage, an early marker for atherosclerosis [24–30] (reviewed in [31, 32]). Cellular and molecular changes induced by radiation exposure occur not only in directly irradiated cells, but also in neighboring non-irradiated cells, a process known as the 'radiation-induced bystander effect' (RIBE) [33, 34]. Transmembrane connexin (Cx) proteins are critical modulators of this process by forming gap junction channels that provide intercellular communication routes between neighboring cells, and hemichannels, that mediate paracrine communication pathway. While understanding of the molecular mechanisms of IR-induced atherosclerosis has increased, the role of intercellular communication, particularly the role of endothelial Cxs and their channels, in the development of radiation-induced atherosclerosis is still poorly defined. Here, we review the role of intercellular communication in radiation-induced atherosclerosis, with the focus on radiation-induced bystander response and a possible role of Cxs in radiation-induced atherosclerosis.

## Intercellular communication in atherosclerosis development and the response to ionizing radiation exposure

### Radiation-induced bystander effect (RIBE)

Biological responses in non-irradiated cells are defined as non-targeted effects [35], which may include genomic instability, bystander effects, and abscopal effects [35–37]. Radiation-induced genomic instability can be observed as a delayed and stochastic appearance of de novo gene mutations, chromosomal aberrations, and reproductive cell death in the progeny of irradiated cells [38]. Bystander effect pertains to cells adjacent to irradiated cells, while the abscopal effect may reach further tissues outside of the irradiated volume, and it relies more on clinical observations in patients receiving radiotherapy [35, 39].

#### RIBE: experimental data

##### In vitro studies

Traditionally, it was accepted that exposure to IR only affected directly irradiated cells. However, in 1992, Nagasawa et al. reported that irradiating 1% of Chinese hamster ovary cells with α-particles led to genetic damage in more than 30% of cells [40]. This observation was later confirmed by others in human fibroblast cells [41]. This means that non-irradiated cells exhibit effects as a result of signals received from adjacent irradiated cells, a process known as radiation-induced bystander effect (RIBE) [34, 42]. Since then, RIBE has been observed in several in vitro studies for different biological endpoints such as cell death, apoptosis, senescence, DNA damage, gene mutations, chromosomal aberrations, genomic instability, cell differentiation, cell cycle distribution, and gene expression (reviewed in [43, 44], and [45]). Bystander effects have mainly been studied in vitro using various techniques (medium transfer, co-culture method and microbeam irradiation which provided clear evidence of RIBE) [43, 46, 47], distinct cell types (normal and cancerous cells) [41, 48, 49], and different culture systems (two and three-dimensional models) [49, 50]. RIBE has been reported to be induced both by high-LET irradiation [47, 51–53], as well as low-LET irradiation after high doses (> 2 Gy) [54–56], medium and low doses of exposure (> 2 Gy) [57–59], utilizing a variety of dose rates [60]. These studies showed that RIBE depends on radiation quality, radiation dose, and dose rate used. RIBE was also reported in response to fractionated irradiation exposure commonly used in radiotherapy, which appears to be dependent on cell type, dose/dose rate, and the interval between fractions [61, 62].

High- and low-LET radiation experimental in vitro evidence overall indicates that the classic bystander effect is detrimental for the cells [38, 51, 63–65]. However, non-classic bystander effects have also been described, reporting increased survival of bystander cells after high dose irradiation [66]. Moreover, the so-called radiation-induced adaptive response, which is the acquisition of radiation resistance induced by priming the cells with low dose irradiation [66, 67] [68, 69], will also act to dampen the bystander effect.

In the context of radiation-induced atherosclerosis, an in vitro study was performed to investigate the crosstalk between irradiated macrophages and human umbilical vein endothelial cells. In this study, 3 Gy γ-irradiated macrophage cells were reported to trigger apoptosis and inflammatory responses in bystander endothelial cells via a p38-dependent pathway [70]. This involved VCAM-1, a pro-inflammatory molecule that enhances monocyte–endothelial adhesion and is a key event in initiating atherosclerosis [71], and MMP-9 that plays an important role in endothelial dysfunction by triggering apoptosis and inflammation [70, 72].

##### Animal studies

Next to the extensive set of in vitro bystander studies, several in vivo animal studies reported RIBE using distinct radiation qualities, radiation doses and dose rates, where oxidative stress, apoptosis, DNA damage, and genetic/epigenetic dysregulations were observed in bystander-shielded organs such as spleen and lung [44, 73–76]. These studies indicated that oxidative stress plays an important role in RIBE in vivo since (pre)treatment of animals with antioxidants significantly reduced DNA damage in shielded regions [75, 76]. Moreover, bystander effects in animals were shown to follow a distinct time scale with consequences persisting for several months after radiation exposure [77]. *Camphausen *et al*.* suggested that in vivo bystander responses may result in an anti*-*tumor effect. They observed that fractionated γ-irradiation of mouse legs, 5 × 10 Gy fractions, and 12 × 2 Gy fractions, slowed down tumor growth in the midline dorsum in a dose-dependent manner, an effect that was mediated by p53 [78]. *Mancuso *et al*.* provided proof-of-principle and mechanistic evidence for RIBE involvement in vivo [79, 80]. They reported tumor induction in bystander-shielded cerebellum of Patched homolog-1 heterozygous radiosensitive mice after X-ray exposure of the lower part of the body. It was furthermore demonstrated that gap junction intercellular communication, together with ATP release and connexin 43 upregulation, were involved in transmission of oncogenic bystander signals to the central nervous system.

##### In humans

Clinically, it is well known that local radiotherapy for different types of cancer may induce distant effects known as “abscopal effects” [42, 81, 82]. The first study (1954) that suggested non-targeted abscopal effects, reported a decrease in the bone marrow cellularity of children that received X-irradiation to their spleen for chronic granulocytic leukemia treatment [83]. Since then, several studies reported abscopal effects in cases where radiotherapy was combined with immune checkpoint inhibitors or immunotherapeutic agents that enhance the immune response in general [84–86]. Abscopal effects may in principle be clinically useful to extend the radiation effects to tumor cells outside the radiation field [81] but they may as well be harmful when reaching distant healthy cells and tissues [5], effectively restricting their application.

The occurrence of secondary cancers in patients treated with radiotherapy, e.g. the development of lung, sarcoma and melanoma cancers after prostate cancer radiotherapy [87, 88], is well established. Another example is the high incidence of secondary lung cancer in ovarian, rectal, and cervical cancer patients treated with radiotherapy [89, 90]. In addition, as previously stated, several studies have reported an increased risk for non-cancerous diseases such as CVD in radiotherapy-treated patients [18]. Due to individualized dose calculation advancements and the prescribed targeted technical approaches in radiotherapy, it is speculated that scattered radiation cannot be the sole trigger explaining the high incidence of secondary cancer as well as non-cancer side effects after radiotherapy. RIBE is postulated to play a role in the development of these post-radiotherapy side effects [91, 92].

#### RIBE: underlying molecular mechanisms and the possible link to atherosclerosis

Although RIBE has improved our understanding of the non-targeted effects after radiotherapy, its molecular mechanisms are complex and not fully understood. Two main routes were reported to underly bystander signals: (i) direct cell-to-cell communication, often mediated by gap junctions and (ii) paracrine release of soluble messengers/factors from directly irradiated cells to the extracellular environment [93, 94]. Paracrine release can be mediated by vesicular release mechanisms in general, exosome release in particular, and by the opening of large pore channels such as connexin hemichannels [95, 96]. Given the prominent role of ATP release in bystander signaling, paracrine purinergic communication through P2X and P2Y receptor families may take a central stage [97–99].

**Fig.1:**
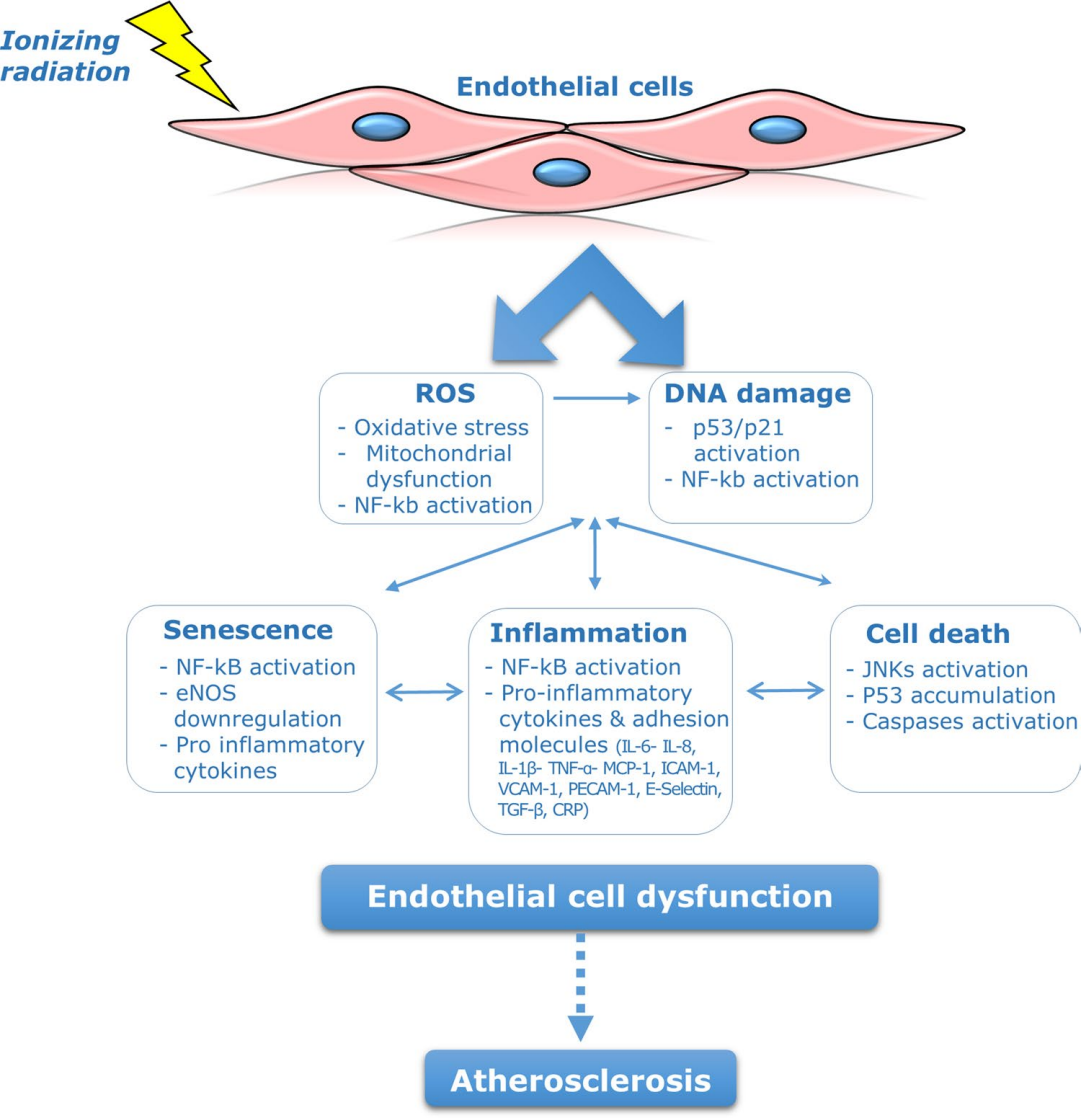
Molecular mechanisms responsible for radiation-induced endothelial cell damage and the development of atherosclerosis

The bystander effect induced by IR involves diverse signaling molecules. Oxidative stress molecules, including reactive oxygen and nitrogen species are the main culprits in activating DNA damage and apoptosis in bystander cells [100, 101]. Oxidative stress also plays a crucial role in the pathophysiology of atherosclerosis, since it is associated with activation of inflammatory and apoptotic pathways that contribute to endothelial cell injury (Fig. 1) [102, 103]. In addition to oxidative stress, IR also triggers the release of various cytokines (e.g. TNF-α, TGF-β, IL-1, IL-2, IL-6, and IL-8) largely—but not exclusively—derived from non-irradiated lymphocytes and macrophages [33, 34, 104, 105]. It is well recognized that atherosclerosis is a chronic inflammatory disease, and elevation of these cytokines in non-irradiated bystander cells may impact the time course of atherosclerotic alterations (Fig. 1) [71]. Therefore, irradiated cancer cells as well as endothelial cells during thoracic radiotherapy, may induce oxidative stress and inflammation in non-irradiated endothelial cells in the cardiovascular system and lead to endothelial injury, which may set the path for atherosclerotic development.

Besides ROS and inflammation, there is substantial evidence that NF-_k_b and MAPK signaling pathways, as well as signaling by intracellular calcium ions (Ca^2+^), cyclooxygenase-2 (COX-2), extracellular ATP, nitric oxide (NO) and p53 protein are involved in bystander effects in non-targeted cells after radiation exposure [33, 63, 106–110]. Epigenetic modulation was also reported to play a role in bystander responses, since changes in DNA methylation and in miRNAs expression have been observed in non-irradiated tissues [77, 111, 112]. In addition, cellular senescence has been proposed to contribute to RIBE, since senescent cells express a particular senescence-associated secretory phenotype that, together with ROS, may activate NF-_k_b leading to a DNA damage response, mitochondrial dysfunction, and inflammation in bystander cells [113–115]. RIBE can also be mediated by the cysteine protease cathepsin B, based on observations in *C. Elegans*, which is regulated by a p53 homologue and acts through insulin-like growth factor receptor signaling resulting in inhibition of cell death and increased embryonic lethality [116].

Several in vitro and in vivo studies have indicated a role for p53 protein, NF-_k_b, and MAPK signaling cascades in the pathogenesis of atherosclerosis (Fig. 1). It was reported that activation of MAPK and NF-_k_b signaling mediates crucial mechanisms involved in the pathogenesis of atherosclerosis such as endothelial cell activation, inflammation, intimal smooth muscle cell proliferation, and T-lymphocyte differentiation [117–121]. It was also reported that foam cell formation in the atherosclerotic lesion depends on JNK2 and p38α MAPK activation [122, 123]. Additionally, it was observed that endothelial-specific NF-_k_b inhibition protected mice from atherosclerosis development by reducing the expression of vascular adhesion molecules, cytokines and chemokines and preventing macrophage recruitment to atherosclerotic plaques, hence strongly reducing atherosclerotic plaque formation [121]. Activation of p53, in response to oxidative stress and DNA damage, was also reported to induce apoptosis and premature senescence in vascular endothelial and smooth muscle cells [124, 125]. Moreover, activation of the NF-κb pathway and sustained inflammation has been illustrated in irradiated arteries of patients that were treated with radiotherapy [126].

**Fig. 2:**
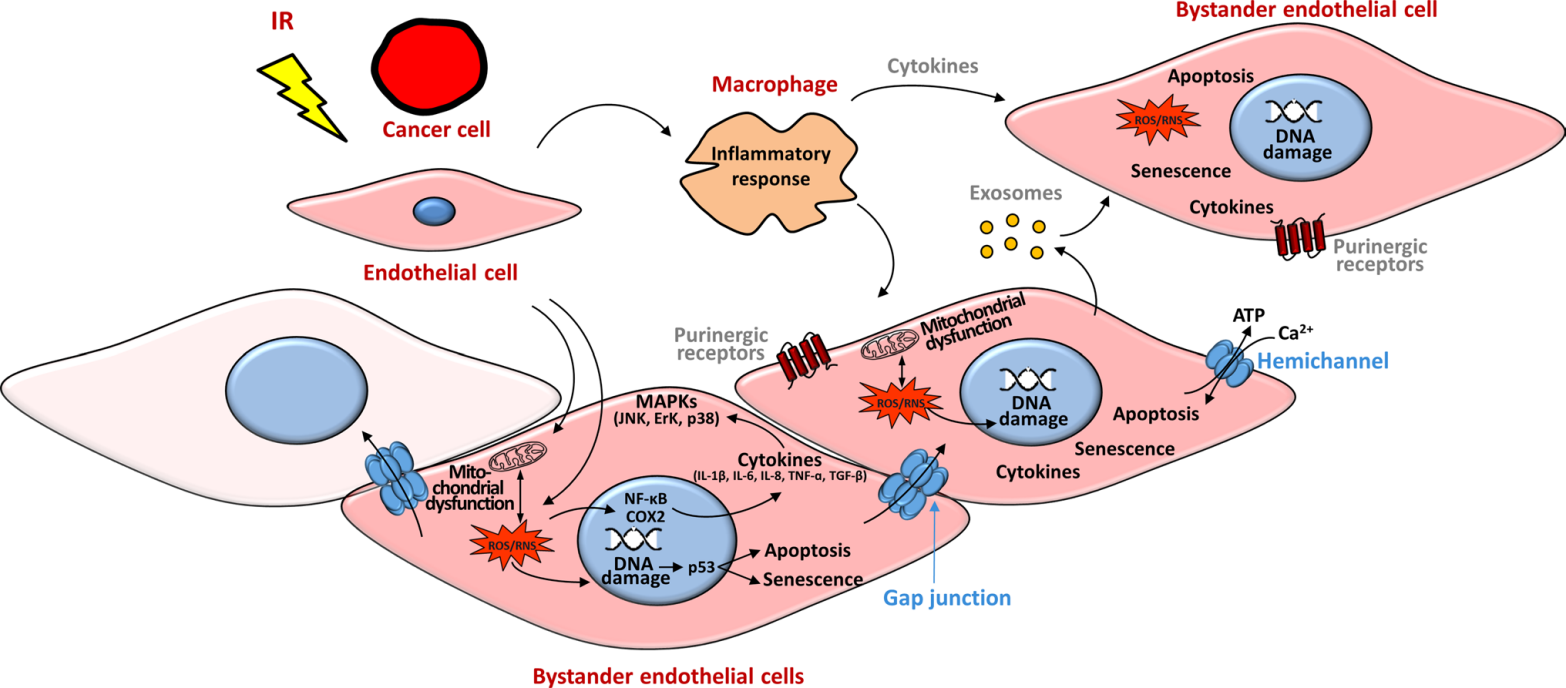
Pathways of radiation-induced signaling potentially leading to bystander endothelial dysfunction

As such, a possible explanation of the cardiovascular effects after thoracic radiotherapy is through the radiation doses received in the heart region, which is estimated to be in the order of 1–20 Gy [5, 18, 127–131]. These doses may damage the endothelium directly to initiate the atherosclerosis process, which may expand via bystander signaling to non-irradiated endothelial cells [55] (Fig. 2).

Irradiated cancer or endothelial cells during thoracic radiotherapy may produce bystander responses to non-irradiated endothelial cells in the cardiovascular system via three main routes: (i) by direct cell-to-cell communication via Cx-based gap junctions, (ii) by paracrine release of soluble factors (e.g. ATP, released via vesicular mechanisms or Cx hemichannels) to the extracellular environment and (iii) by exosomes, which may use Cxs to interact with their targets [132]. Not all the cells are affected by bystander signaling (fade pink cell). Macrophages may be important mediators in the bystander response, by regulating cytokine release to bystander cells. Reactive oxygen and nitrogen species (ROS/RNS), signaling cyclooxygenase-2 (COX-2) together with signal transduction through p53, MAPKs and NF-κb may be involved in bystander responses in non-targeted endothelial cells after radiation exposure. Eventually, these signaling molecules may participate in endothelial cell dysfunction by triggering DNA damage, apoptosis, senescence, mitochondrial dysfunction and inflammation.

### Intercellular communication and the role of connexins in atherosclerosis

#### Connexins and their channels

**Fig. 3:**
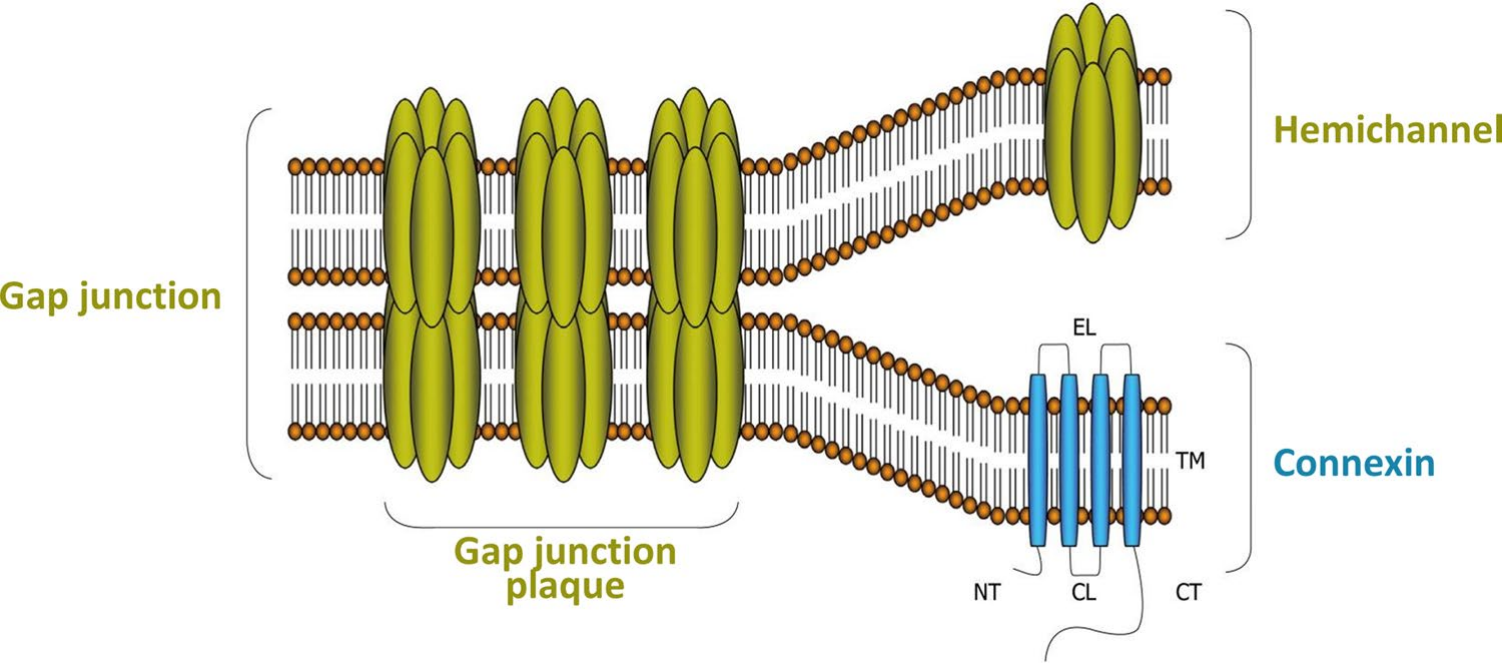
Molecular architecture of Cxs, hemichannels, and gap junctions. Cx proteins consist of four TMs, two ELs, one CL and a cytoplasmic NT and CT. Gap junctions are composed of 12 Cx proteins, organized as two hexameric hemichannels of two apposed cells. *Cx *connexin, *TM* transmembrane domain, *EL* extracellular loop, *CL* cytoplasmic loop, *NT* NH_2_ terminus, *CT* COOH terminus [133]

As delineated before, gap junctions and hemichannels play an important role in communicating bystander signals. Both gap junctions and hemichannels are composed of a transmembrane protein called connexin (Cx) (Fig. 3). There are 21 human Cx isoforms (20 in the murine genome), which exist in either phosphorylated or non-phosphorylated forms. The nomenclature of Cx is based on their molecular weight, which ranges from 25 to 62 kDa [34]. Each Cx protein consists of four transmembrane domains (TM1-4). These domains are connected by two extracellular loops (EL) that regulate docking processes and cell–cell recognition. The proteins have a cytoplasmic carboxy-terminal tail (CT), amino-terminal tail (NT), and a short cytoplasmic loop (CL) linking TM2 and TM3. Six Cx proteins oligomerize to form a hemichannel; two opposed hemichannels from adjacent cells form a gap junction channel by the interaction of conserved domains on the extracellular loops of hemichannels [133]**.** The life cycle of Cxs is characterized by various steps, including Cx trafficking to the cell surface, hemichannel formation, gap junction assembly, gap junction plaque formation, and closure of the cycle by gap junction disassembly through internalization and degradation (Fig. 3) [134]. Cxs are expressed in a tissue- and cell-specific manner, with Cx43 being the most abundant and widespread isotype in mammals. Cx43 is also a major isotype in the cardiovascular system and is especially abundant in ventricular cardiomyocytes [135, 136]. Apart from the cardiovascular system, Cx43 has also major functions in brain astrocytes and vascular endothelial cells, the kidneys and the reproductive organs [137].

The physiological role of Cxs has been demonstrated by gene knockout studies and human diseases associated with Cx mutations. For instance, Cx43-knockout mice die at birth due to cardiac malformation, illustrating the crucial role of this specific isotype in development [138]. Moreover, Cx43 deletion in mice has a major impact on the gene network and dysregulates genes involved in the differentiation and function of vascular cells and in vasculogenetic/angiogenetic signaling pathways, therefore interfering with normal development of coronary arteries [139, 140]. The deletion of one allele of Cx43 in Cx40-knockout mice exhibited cardiac malformations and led to neonatal death [141]. The simultaneous ablation of Cx37 and Cx40 caused vascular abnormalities in intestine, skin, stomach, lung, and testis [142]. Polymorphisms of Cx37 were reported to be associated with coronary artery disease, including atherosclerosis, and myocardial infarction [143–145]. These observations highlight the role of Cxs in the development of the vasculature and for maintaining vascular homeostasis.

Next to the physiological role of Cx proteins, cell–cell communication via gap junctions and hemichannels may become disturbed as cause or consequence under pathological conditions [34]. Gap junctions permit passive diffusion of atomic ions (e.g. Ca^2+^, Na^+^, Cl^−^ and K^+^) and of small (molecular weight below ~ 1.5 kDa) hydrophilic molecules (e.g. ATP, glucose, glutamate, IP3 and glucose) and other second messenger molecules between adjacent cells [146]. Gap junctions are usually open to promote crosstalk between the cells and to facilitate the propagation of chemical and electrical signals between the cytoplasm of neighboring cells, thereby serving as a key mechanism in the synchronization of physiological signals [146–148]. In the heart, gap junction channels facilitate action potential conduction along conductive tissues as well as between cardiomyocytes, and synchronize the atrioventricular contraction cycle [148, 149]. In the vascular wall, gap junctions in endothelial cells, smooth muscle cells and between these cells facilitate electrical and chemical signaling, thereby coordinating vasoactive responses [148–150].

Unlike gap junctions, plasma membrane hemichannels may facilitate cell–cell communication via paracrine signaling. Hemichannels normally remain closed to prevent leakage of substances that could deplete the cell from crucial metabolites or harm neighboring cells [151]. Opening of hemichannels has been demonstrated to play a role in physiology, e.g. in bone where they promote periosteal remodeling processes [152] (reviewed in [153]), or in brain where they may contribute to gliotransmitter release, either as channel facilitating gliotransmitter passage, or as a channel that allows (non-selective) Ca^2+^ entry triggering release through other pathways [154]. However, most of the evidence currently available links hemichannel opening to pathological conditions [135, 138, 155–159]. Hemichannels can open in response to several signals, including membrane potential changes, intracellular Ca^2+^ elevation, mechanical stimulation and stress-associated stimuli such as oxidative stress, ischemic or pro-inflammatory conditions and radiation exposure [95, 155, 160–164]. Once hemichannels are open, evidence from various experimental approaches indicates they facilitate the passage and loss of intracellular prostaglandin E2, NAD^+^, IP3, glutathione, ATP and K^+^, and the entry of Ca^2+^ and Na^+^ [151, 155, 160–162, 165]. This may lead to downstream responses including NO production, cell proliferation, cell death, NLRP3 inflammasome pathway, and inflammation [34, 95, 134, 166–172]. Some of the released messengers, e.g. ATP, may function as a paracrine messenger of bystander signaling thereby expanding radiation-induced biological effects [34, 55, 167]. In addition, open hemichannels may allow direct passage of ROS because of the small size (< 1000 Da) of most oxidative stress-inducing molecules and can cause cellular injury or death [160]. Increased hemichannel opening activity was observed in several inflammatory diseases and blocking these channels inhibited the inflammation [171, 173–177]. In addition, specific blocking of hemichannels was suggested to improve gingival wound healing [178], decrease amounts of liver lipids and inflammatory markers in non-alcoholic steatohepatitis in mice [179], reduce cardiac arrhythmogenesis in Duchenne muscular dystrophy mice [180, 181] and in MYL-4-related atrial cardiomyopathy and fibrillation [180, 182–184], reduce dopamine neuron loss and microglial activation [185], provide neuroprotection in stroke [186], and protect against seizures in rodents [187].

#### Connexins and atherosclerosis

The endothelial cells of the vascular system’s major arteries express three prominent Cx isotypes, namely Cx37, Cx40 and Cx43, with Cx43 being the most abundant isoform. In addition, Cx45 is expressed in the endothelium of the large arteries only. The smooth muscle cells that surround the vascular endothelial cells present mainly Cx43, Cx40 and Cx45. Five different Cx isotypes (Cx31.9, Cx37, Cx40, Cx43, and Cx45) are expressed in the heart, with Cx43 being the predominant connexin in ventricular myocardium. The turnover of these Cx proteins is very fast with a half-life ranging from one to five hours. Consequently, Cx proteins can quickly respond to several conditions due to the plasticity of their expression and the fast dynamics of the formed hemichannels and gap junctions [188]. Therefore, changes in Cx expression may directly be translated to changes in bystander response (Fig. 2).

##### Proatherogenic Cxs

There is growing evidence that Cx proteins play an important role in atherosclerosis development. Cx43 is normally absent in the aortic endothelium of healthy individuals; however, it can be detected at the plaque shoulder region, which is located close to areas of plaque necrosis, a region known to be prone to plaque rupture, and at branching sites of the arterial tree, which are highly susceptible to atherosclerosis development [189, 190]. High Cx43 expression was reported at regions of disturbed blood flow in rat aortic endothelial cells, and increased Cx43 expression was also observed in various in vivo studies using a model that simulates human arterial shear stress [191–194]. It is known that a hemodynamic-shear stress environment plays a critical role in atherogenesis by promoting a pro-inflammatory phenotype in the endothelium [195]. Upregulated Cx43 gap junctions between intimal smooth muscle cells were also reported in human coronary artery specimens at regions of intimal thickening and early atheromatous lesions compared to healthy vessels [196]. It has been reported that endothelial Cx43 expression regulates monocyte‑endothelial adhesion, which is a crucial initiator of atherosclerosis development, as increased Cx43 expression enhanced the expression level of cell adhesion proteins, including VCAM-1 [197]. Decreased Cx43 expression was reported to reduce atherosclerotic lesion formation as well, and to reduce inflammation in low-density lipoprotein receptor-deficient mice, hence to reduce atherosclerosis progression by half [198, 199]. Collectively, these observations suggest that Cx43 is a proatherogenic protein that may stimulate atherosclerosis development.

Next to the alterations in Cx43 expression and gap junction function during atherosclerosis development, dysfunctional hemichannels have also been suggested to take part in the process. Cx43 hemichannel activity was significantly increased in endothelial cells exposed to pro-inflammatory conditions (IL-1β/TNF-α) and high glucose levels, known to cause vascular dysfunction, leading to increased ATP-dependent Ca^2+^ dynamics [200]. In this study, they showed that inhibiting Cx43 hemichannels prevented endothelial ATP release [200] which induces vascular inflammation and atherosclerosis in mice via the activation of purinergic Receptor Y2 [201].

##### Atheroprotective Cxs

In contrast to Cx43, Cx37 and Cx40 proteins play an atheroprotective role. Endothelial Cx37 and Cx40 are almost absent in advanced atherosclerotic plaques while present in healthy arteries [190, 202, 203]. Besides, it has been reported that Cx40-deficient mice, with a coincident reduction in Cx37, are associated with lower eNOS expression levels in the aortic endothelium, leading to a reduced NO release and smaller endothelium-dependent relaxations of the aorta [204]. Therefore, decreased NO bioavailability has been linked to an increased susceptibility to atherosclerosis [205]. In another study, it was observed that ApoE-/-mice lacking Cx37 gene (GJA4) developed more aortic lesions than ApoE-/-mice that express Cx37 at normal levels [206]. In vivo and in vitro approaches showed increased recruitment of monocytes and macrophages to the atherosclerotic lesions and increased leucocyte transmigration. Therefore, they suggested that Cx37 may inhibit atherosclerosis development by tempering leukocyte adhesion [206]. Additionally, a downregulated endothelial Cx37 was observed in response to shear stress, which is known to induce endothelial dysfunction [207]. A recent study demonstrated decreased Cx37 expression in response to oxidized LDL, a major component of hyperlipidemia and contributor to endothelial injury, in the human monocyte cell line THP-1, which was associated with increased monocyte–endothelial adhesion, thus potentially promoting atherosclerosis development [208].

Related to Cx40, it was observed that endothelial-specific deletion of Cx40 increased CD73-dependent leukocyte–endothelium adhesion, thereby potentially promoting the atherosclerotic process [209]. This study further reported that Cx40-mediated gap junctional communication between endothelial cells generated anti-inflammatory signals that may contribute to a quiescent non-activated endothelium, thus protecting against atherosclerosis. A recent study reported lowered Cx40 expression in mice carotid arteries under oscillatory shear stress, which was associated with NF-_k_B activation [210]. They further revealed a novel function of I_k_Bɑ-Cx40 interaction involved in controlling NF-_k_B-mediated endothelial cell activation by shear stress in atherogenesis.

Despite these interesting observations discussed above, the relation between altered Cx expression and the atherosclerotic process is not entirely clear yet, especially with respect to the question whether Cxs are causally or consequentially linked to the atherosclerotic process. For instance, a study reported that TGF-β, a major inflammatory component in the atherosclerotic process, induced upregulation of Cx43 in endothelial cells [211]. In addition, it was found that the atherosclerosis-associated inflammatory markers, TNF-ɑ and INF-γ, increased Cx43 expression in monocytes [212]. TNF-ɑ treatment also increased Cx43 at the mRNA level, while it reduced Cx37 and Cx40 mRNA in human umbilical vein endothelial cells (HUVEC) [213]. In line with this, endothelial Cx40 deletion in mice induced spontaneous atherosclerotic plaques in the aortic sinus, without introducing a high-cholesterol diet [209], which support the important role of Cx proteins in initiating the atherosclerotic process.

### Response of connexins and their channels to ionizing radiation exposure

Cx expression and channel activity have been shown to rapidly change upon intra- and extracellular modifications or in response to stimuli, including ionizing radiation, thereby changing the extent of intercellular communication [188, 214–218]. Alterations in Cx43 expression were reported in response to low or high doses of IR, and high-LET as well as low-LET radiation exposure. For instance, upregulated Cx43 expression was reported after exposure to 10 mGy of α-particles as well as 4 Gy of γ-rays in normal human skin fibroblasts, mouse embryo fibroblasts, and rat liver epithelial cells, which was associated with a corresponding increase in gap-junctional intercellular communication [219]. Upregulation of Cx43 was also observed upon in vivo exposure of cardiac myocytes to heavy-ion irradiation [220–222]. Gamma-ray radiation was furthermore found to induce Cx43 upregulation in mouse skin [223] and human neonatal foreskin fibroblasts irradiated with single low doses of IR [216]. Similarly, X-rays (5 Gy) increased Cx43 gene expression and protein level in the bEnd3 endothelial cell line derived from mouse brain capillaries. However, umbilical vein hybrid endothelial cells (EA.hy926) responded oppositely, displaying transient Cx43 downregulation after 5 Gy X-ray exposure, suggesting that Cx43 modulation in response to radiation exposure may be cell-line dependent [224]. A recent study also observed that low doses of γ-rays (10–20 cGy) enhanced Cx43 expression and gap-junctional coupling in U87 glioma cells, and induced Cx43 overexpression in tumor cells of varying origin [215]. Interestingly, B16-melanoma cells showed Cx43 hemichannel opening in response to 0.5 Gy γ-rays, as concluded from ATP release measurements [95]. The mechanisms responsible for Cx43 alteration in response to IR are not known yet; post-irradiation oxidative stress has been proposed [219], and the nuclear factor of activated T cells (NFAT) together with activator protein (AP1) transcription factors were shown to be responsible for the major activation of the Cx43 promoter in response to gamma irradiation [216].

**Fig. 4:**
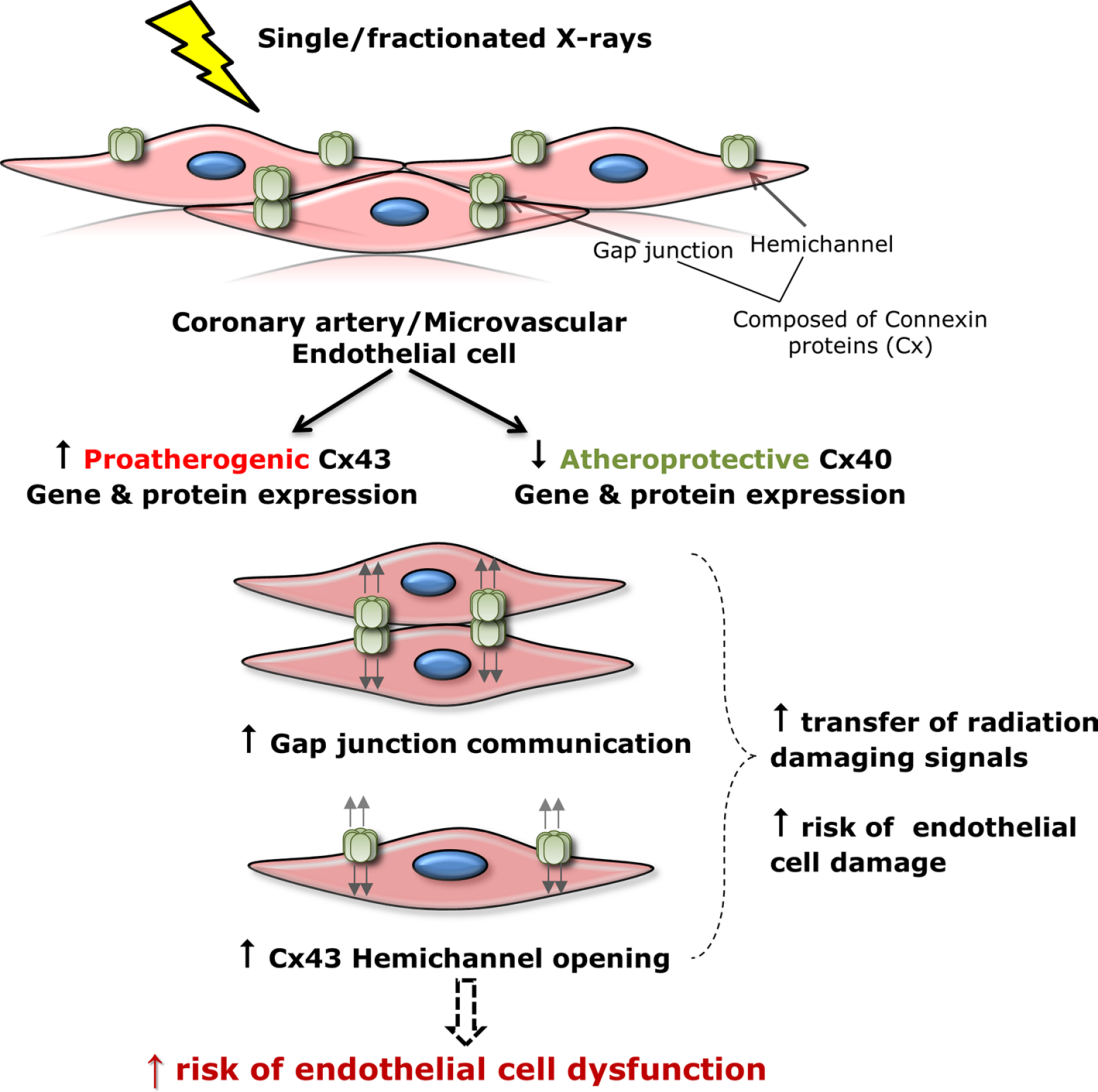
Summary scheme of X-ray effects on endothelial connexins (Cxs) and their channels [164]

Although there is growing evidence indicating the sensitivity of Cx43 in response to radiation exposure, there is lack of data regarding Cx modulation in endothelial cells, the primary target site for atherosclerosis development, in response to IR exposure. Moreover, knowledge on radiation-induced alterations in endothelial Cx37 and Cx40 is very limited. We found that exposure of immortalized coronary artery and microvascular endothelial cells to low and high doses of X-rays, delivered as a single or fractionated dose, dose-dependently decreased atheroprotective Cx37 and Cx40, while increasing proatherogenic Cx43, over a 14 day observation period (Fig. 4). Single and fractionated irradiations were also shown to induce an increase in gene expression and protein levels of the proatherogenic Cx43 in both coronary artery and microvascular endothelial cells, which was persistent until 14 days after exposure [164]. Similar alterations in Cx expression levels have been reported in the literature in endothelial cells covering atherosclerotic plaques [134, 189, 190, 202]. Thus, Cx alterations observed in our study may promote susceptibility to atherosclerosis after IR exposure. Next to Cx alterations, single and fractionated exposures increased gap junctional communication and induced acute and long-lived Cx43 hemichannel opening persisting over 72 h after IR in coronary artery and microvascular endothelial cells [164]. As delineated before, excessive hemichannel opening is considered a pathological condition, since it results in loss of cell-essential metabolites and ATP leakage that act in a paracrine manner on surrounding cells. In turn these messengers, with ATP as the principle actor, can activate downstream cellular processes including propagating intercellular Ca^2+^ waves, oxidative stress responses, apoptosis, NLRP3 inflammasome pathway activation and inflammation (50–52), which are to known to be involved in the pathogenesis of radiation-induced atherosclerosis. Moreover, radiation-induced increased endothelial gap-junctional coupling and hemichannel function may spread radiation damaging responses to neighboring cells, possibly amplifying endothelial cell damage (Fig. 2) [46, 55, 166, 214, 219, 225–227]. Together, these findings suggest a possible mechanism of radiation-induced atherosclerosis (Fig. 4), which may guide us in further improving our understanding of Cx proteins as a potential target to prevent radiation-induced cardiovascular complications. Interestingly, we found that the Cx43 hemichannel-inhibiting peptide TAT-Gap19 mitigated radiation-induced endothelial cell damage by reducing oxidative stress, cell death, premature cell senescence and pro-inflammatory and pathological factors like IL-1β, IL-8, VCAM-1, MCP-1 and endothelin-1 in immortalized coronary artery and microvascular endothelial cells [228]. Therefore, targeting Cx43 hemichannels may hold potential to protect against radiation-induced endothelial cell damage.

## Conclusion

Growing evidence indicates an excess risk of radiation-related side effects such as late occurring cardiovascular diseases, especially atherosclerosis. However, the exact pathophysiological mechanisms underlying radiation-induced atherosclerosis are not completely understood, possibly resulting in improper radiation protection. Ionizing radiation induces cellular effects such as DNA damage, oxidative stress, inflammation, apoptosis, and premature cell senescence which may induce endothelial cell dysfunction, a primary marker for atherosclerosis. Intercellular communication through gap junctions and hemichannels, which propagate radiation-induced bystander effects, may modulate the endothelial response to ionizing radiation, and therefore the atherosclerotic process. Although Cxs were shown to be altered by radiation exposure and to play a role in atherosclerotic development, current evidence linking the two processes is still lacking. More studies are needed to clarify the role of Cxs and their channels in radiation-induced atherosclerosis, possibly leading to new opportunities for targeting connexins and its channels.

## Abbreviations

ATP: Adenosine triphosphate
Cx: Connexion
CVD: Cardiovascular diseases
CL: Cytoplasmic loop
CT: C-terminal tail
COX-2: Cyclooxygenase-2
DNA: Deoxyribonucleic acid
DSB: Double-strand breaks
eNOS: Endothelial nitric oxide synthase
EL: Extracellular loop
Gy: Gray
IR: Ionizing radiation
IP3: Inositol triphosphate
IL: Interleukin
JNK: C-jun N-terminal kinase
kDa: Kilo-Dalton
LDL: Low-density lipoprotein
MAPK: Mitogen-activated protein kinase
MCP-1: Monocyte chemotactic protein-1
NF-κB: Nuclear factor kappa-light-chain-enhancer of activated B cells
NO: Nitric oxide
NT: NH_2_ termini
RIBE: Radiation-induced bystander effect
ROS: Reactive oxygen species
RNS: Reactive nitrogen species
SSB: Single-strand break
TGF: Transforming growth factor
TNF: Tumor necrosis factor
TM: Transmembrane domain
VCAM-1: Vascular cell adhesion molecule 1
